# The role of NTHi colonization and infection in the pathogenesis of neutrophilic asthma

**DOI:** 10.1186/s12931-020-01438-5

**Published:** 2020-07-03

**Authors:** Jing Zhang, Zhenxing Zhu, Xu Zuo, He Pan, Yinuo Gu, Yuze Yuan, Guoqiang Wang, Shiji Wang, Ruipeng Zheng, Zhongmin Liu, Fang Wang, Jingtong Zheng

**Affiliations:** 1 Department of Intensive Care Unit, First Hospital of Jilin University, Changchun, 130021 China; 2 Department of Pathogen Biology, College of Basic Medical Sciences, Jilin University, Changchun, 130021 China; 3 Department of Hematology and Oncology, China-Japan Union Hospital of Jilin University, Changchun, 130033 China; 4 Department of Interventional Therapy, First Hospital of Jilin University, Changchun, 130021 China; 5 Key Laboratory of Zoonosis, Ministry of Education, College of Veterinary Medicine, Jilin University, Changchun, 130062 China

**Keywords:** Neutrophilic asthma, Nontypeable *Haemophilus influenzae* (NTHi), Oxidative stress, Th17/Treg imbalance, Corticosteroid-resistant

## Abstract

Asthma is a complex heterogeneous disease. The neutrophilic subtypes of asthma are described as persistent, more severe and corticosteroid-resistant, with higher hospitalization and mortality rates, which seriously affect the lives of asthmatic patients. With the development of high-throughput sequencing technology, an increasing amount of evidence has shown that lower airway microbiome dysbiosis contributes to the exacerbation of asthma, especially neutrophilic asthma. Nontypeable *Haemophilus influenzae* is normally found in the upper respiratory tract of healthy adults and is one of the most common strains in the lower respiratory tract of neutrophilic asthma patients, in whom its presence is related to the occurrence of corticosteroid resistance. To understand the pathogenic mechanism by which nontypeable *Haemophilus influenzae* colonization leads to the progression of neutrophilic asthma, we reviewed the previous literature on nontypeable *Haemophilus influenzae* colonization and subsequent aggravation of neutrophilic asthma and corticosteroid resistance. We discussed nontypeable *Haemophilus influenzae* as a potential therapeutic target to prevent the progression of neutrophilic asthma.

## Background

**Fig. 1:**
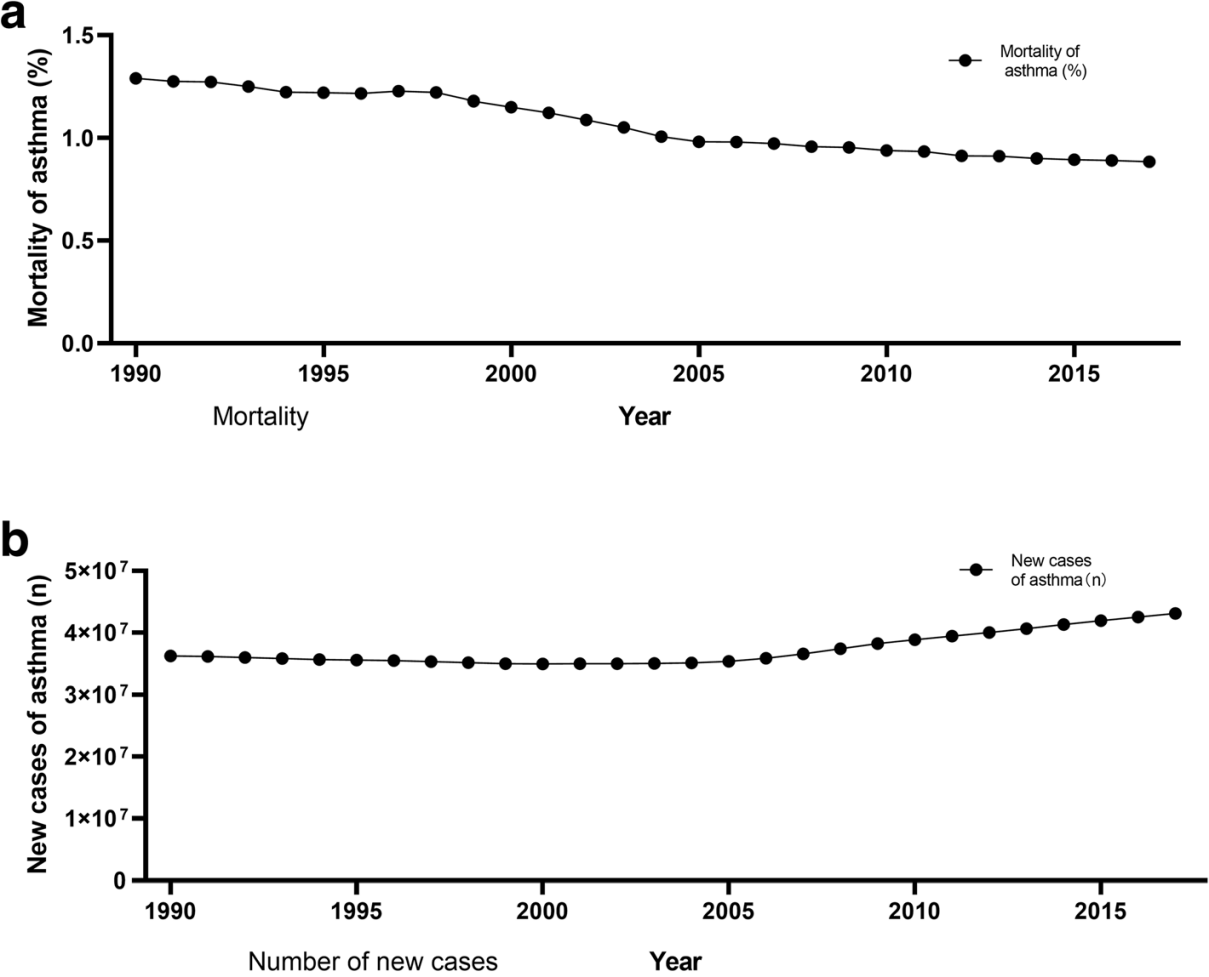
Global mortality of asthma (**a**) and the number of new cases of asthma (**b**) between 1990 and 2017 among all ages. Created with data from the Global Burden of Disease Study 2017 (GBD 2017) Results

Asthma is a complex airway inflammatory disease. It has been reported that there are more than 300 million asthma patients worldwide. The incidence of asthma varies greatly among different countries and regions. In recent decades, the mortality rate of asthma has declined significantly worldwide (Fig. 1a), mainly due to the widespread use of inhaled corticosteroids (ICS). However, the number of new cases of asthma is still increasing (Fig. 1b). According to a prediction from the Global Initiative for Asthma (GINA), 400 million people worldwide will suffer from asthma by 2025. Asthma is considered to be a major cause of disability, substantial medical expenditures and preventable death.

In the past, asthma was too often viewed as a monolithic entity and was known as a type 1 hypersensitivity disease with eosinophilic bronchitis. The Th2 response plays a significant role in asthma, leading to interleukin-4 (IL-4), IL-5, and IL-13 production, IgE-mediated responses, mucus secretion and airway hyperreactivity (AHR) [1]. These are classical explanations of asthma. The view of asthma as a single entity model is now obsolete because people have a better understanding of the heterogeneity of asthma. The latest definition of asthma is based on a history of respiratory symptoms, such as wheezing, shortness of breath, chest tightness and cough, which vary with time and have varying intensity, and variable expiratory airflow restrictions. This clinical definition focuses on variable respiratory symptoms and variable airflow limitations, which are two key features needed for an asthma diagnosis, rather than the pathological and physiological characteristics of asthma that were previously used [2].

Asthma can be divided into subtypes, which include eosinophilic asthma, neutrophilic asthma, mixed granulocytic asthma and paucigranulocytic asthma, according to the sputum cell count and classification [3]. While eosinophilic inflammation has been considered to be the hallmark of airway inflammation in asthma [2], it is present in only 50% of asthmatic patients [4]. Compared with eosinophilic asthma, neutrophilic asthma is described as persistent, more severe and corticosteroid-resistant. Although neutrophilic asthma accounts for only 5–20% of all asthma cases, it consumes more than 50–80% of the medical resources related to asthma and has higher hospitalization and mortality rates, which seriously affect the lives of asthmatic patients and have also become a substantial burden on society and the public health system [5, 6]. Therefore, neutrophilic asthma is currently the most perplexing and challenging presentation of “asthma”.

Many theories have been proposed to explain the rising incidence of asthma. The most striking is Strachan’s hygiene hypothesis, which holds that the increasingly clean and sterile environment in modern life promotes the development of many diseases, including asthma, suggesting that some bacteria may play a protective role in the occurrence of asthma [7]; however, animal model studies support the role of the microflora in the development of asthma and atopic diseases [8]. In recent years, with the development of microbial identification technologies (culture-independent technology), the identification of microbial species, especially bacteria, has become increasingly sensitive. The overall composition of microbial communities has been analyzed in detail [9]. Studies have confirmed that bacterial diversity and microbial community composition are related to the degree of AHR. The airway microbial diversity of patients with neutrophilic asthma decreases significantly. The increase in members of *Haemophilus influenzae* is associated with asthma and is related to the severity of this disease [10, 11].

Nontypeable *Haemophilus influenzae* (NTHi) is a noncapsular strain of *Haemophilus influenzae* and one of the main pathogenic bacteria of the respiratory tract. With the wide application of the *Haemophilus influenzae* type b conjugate vaccine [12], the incidence of NTHi is increasing annually [13]. Current studies have shown that NTHi is one of the most common bacteria isolated from asthmatic patients and may play an important role in the progression and worsening of asthma [14], especially neutrophilic asthma [15]. In this review, we focus on the structure and pathogenicity of NTHi and describe recent insights into the role of NTHi in neutrophilic asthma exacerbation. We will discuss the potential of treating NTHi infections and improving airway flora imbalances as a strategy for treating neutrophilic asthma and preventing its onset.

### NTHi structure and pathogenicity

*Haemophilus influenzae* strains are divided into those with a capsule (e.g., the type b form) and those without a capsule (such as NTHi). The encapsulated strains primarily play roles in systemic infections in conditions such as meningitis, while NTHi strains rarely cause disease outside the respiratory tract and can be considered the main pathogen of the respiratory mucosa.

There is abundant evidence that NTHi is an intracellular pathogen. NTHi has a variety of structures, such as lipooligosaccharides (LOSs), outer membrane proteins, adhesions, pili, Hia and hap proteins, which can inhibit ciliary function and bind to mucus to help NTHi adhere to the respiratory mucosa [16–18]. This is the first step of NTHi colonization. At the same time, NTHi also has an excellent ability to escape mucosal immune mechanisms, including proteases, microflora and phase changes and antigenic drift [19–22]. After evading host mucosal immunity, NTHi can use its invasive ability to enter local respiratory tissues and survive in cells.

The mechanism through which NTHi enters host cells is very complex and includes actin, tubulin, the formation of a lamellar foot and microvilli, and the ingestion of bacteria into vesicles [23]. The most effective entry mechanism seems to be through the interaction of phosphatidylcholine residues on NTHi oligosaccharides with human platelet activating factor receptor (PAFR), as blocking PAFR with specific inhibitors can reduce the invasion of NTHi by more than 90% [24]. Other possible host pathways utilized by pathogens include macropinocytosis, phagocytosis, receptor-mediated endocytosis, lipid raft-mediated endocytosis, autophagy, secretion, transcytosis, and paracytosis [25]. The target cells of NTHi are mainly macrophages and airway epithelial cells [25], in which NTHi can escape the killing effects of antibiotics and bactericidal antibodies [23]. In addition, NTHi can evade the killing effects of the complement system in some unique ways, such as altering LOSs, sialic acid and other outer membrane proteins to suppress complement system activation, enhancing the effect of complement activation inhibitors such as C4 binding protein and factor H [26], inhibiting the function of lymphocytes [27], inhibiting the effects of IgA (important antibodies for killing NTHi via mucosal immunity) and downregulating the expression of IgA. Through these structures and functions, NTHi creates an opportunity to settle in the human respiratory tract for a long time without being cleared by the host immune system.

NTHi is a bacterium that is present in the nasopharynx of most healthy adults, and in this situation, it appears to be a commensal organism [28]. NTHi may also spread to the lower respiratory tract and has been suggested to be associated with many important diseases, such as otitis media, lower respiratory tract infections, the exacerbation of chronic obstructive pulmonary disease (COPD), bronchiectasis and cystic fibrosis [12, 26, 28]. Accumulating evidence suggests that the development of asthma is related to NTHi colonization. Specifically, NTHi infection in early life is closely related to the occurrence of childhood asthma. Recent animal experiments have confirmed this hypothesis. Infection with NTHi on the third day after birth in mice significantly increases the possibility of developing or aggravating asthma in infancy [4]. Hilty and colleagues have come to a similar conclusion for adults and found that *Haemophilus* is more likely to be present in the lower respiratory tract of adults with asthma than in patients with normal lung function [10]. In fact, NTHi is one of the most common bacteria isolated from the airway of patients with neutrophilic asthma [27]. However, it is not clear what role NTHi plays in the occurrence and deterioration of neutrophilic asthma. This review will summarize the previously published mechanisms through which NTHi may lead to the occurrence and aggravation of neutrophilic asthma and will speculate on the relationship between NTHi and neutrophilic asthma.

### Potential mechanisms by which NTHi leads to asthma

#### Hypersecretion of mucus

**Fig. 2:**
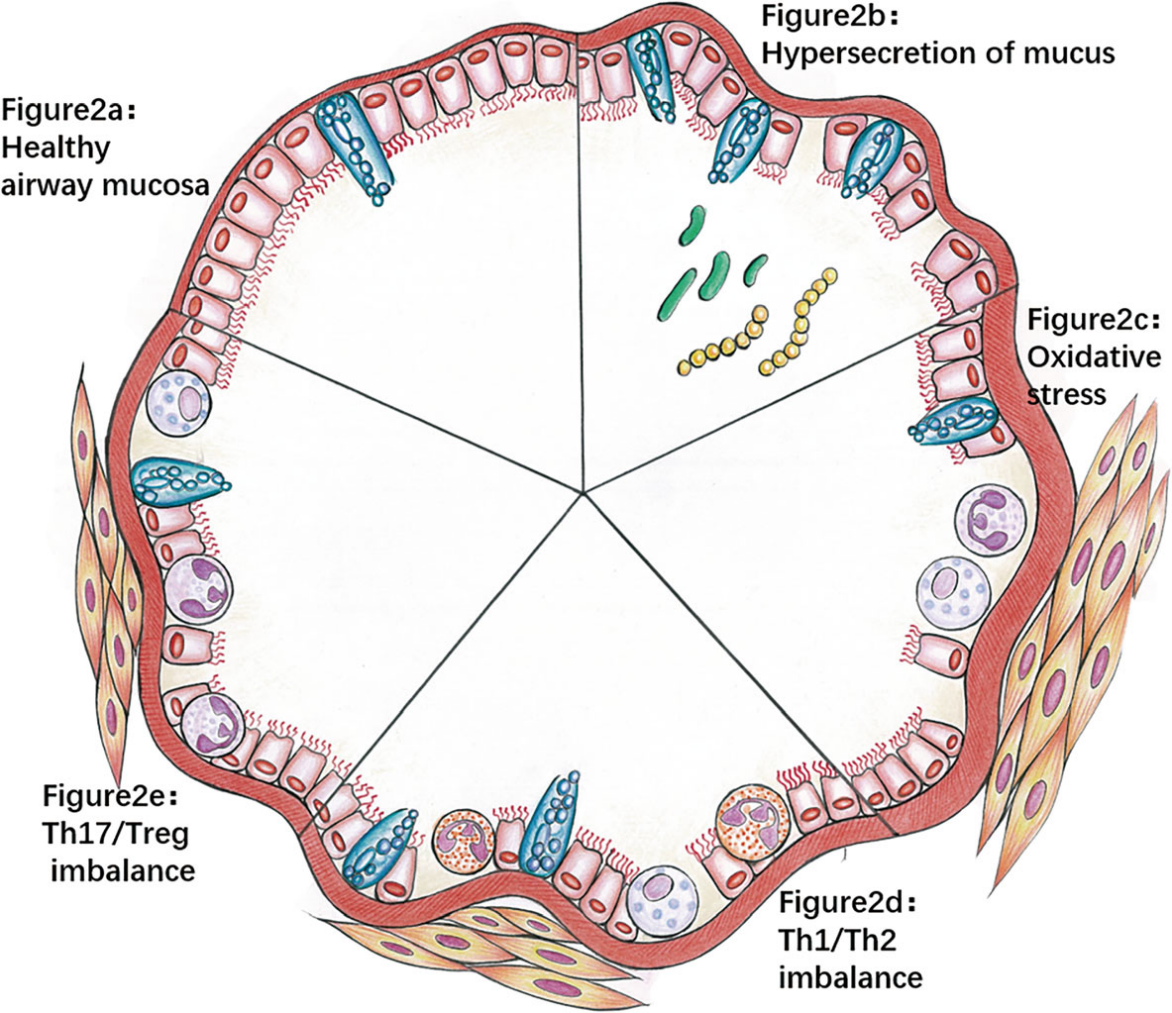
Potential mechanisms by which NTHi leads to asthma. Healthy airways (**a**) have no inflammatory cell infiltration, and goblet cells secrete mucus to lubricate the airway. After NTHI infection: When large amounts of mucus are secreted (**b**), the airway is narrow and obstructed, and other bacteria may cause repeated infections, resulting in airway damage. When oxidative stress occurs, (**c**) neutrophils and mast cells accumulate in the airway, and mucus secretion increases, which is accompanied by airway remodeling and severe airway injury. When Th1/Th2 imbalance (**d**) occurs, the number of eosinophils and mast cells increases and mucus secretion increases, which is accompanied by airway remodeling and airway injury. When Th17/Treg imbalance occurs (**e**), a large number of neutrophils accumulate in the airway, and mucus secretion increases, which is accompanied by airway remodeling and airway injury

Mucus secretion and clearance in the normal airway mucosa are in a dynamic balance, which means that mucus not only protects the airway but also clears dust and pathogens in the inhalation airway without causing airway obstruction (Fig. 2a). The hypersecretion of mucus is one of the manifestations of acute asthma attacks and is one of the characteristics of airway remodeling in neutrophilic asthma. McCann et al. [4] observed that previously NTHi-colonized, allergen-sensitized mice secreted increased amounts of mucus into the airways [14]. Researchers have studied the mechanism by which NTHi infection induces excessive mucus secretion in airway epithelial cells. NTHi can strongly promote transcription of the mucin MUC5AC (MUC5AC is one of the main components of mucus) by upregulating the p38 mitogen-activated protein kinase (MAPK) pathway [29]. In addition, the secretion of MUC2 in asthma is also significantly increased, and because MUC2 is highly insoluble, even a small amount of MUC2 may lead to excessive airway mucus viscosity and airway obstruction in asthma [30]. The mechanism of increased MUC2 secretion caused by NTHi infection is related to the transforming growth factor-beta-Smad signaling pathway in cooperation with nuclear factor-kappa B (NF-κB) [31]. The oversecretion of airway mucus can lead to airway stenosis, airflow obstruction and repeated respiratory tract infections (Fig. 2b). This may be one of the main mechanisms by which NTHi causes neutrophilic asthma to worsen.

#### Oxidative stress

Oxidative stress refers to the damage that occurs when reactive oxygen species (ROS) exceed the host’s antioxidant defenses. Oxidative stress is one of the fatal causes of airway damage in chronic inflammatory diseases, such as COPD, bronchiectasis and neutrophilic asthma. A large amount of evidence has shown that oxidative stress is highly involved in the pathogenesis of human asthma and plays a key role in AHR, neutrophilic inflammation, airway remodeling, and even corticosteroid resistance [32]. Nuclear factor erythroid 2-related factor 2 (Nrf2) is involved in a classical antioxidant pathway. When Nrf2 gene dysfunction occurs, animal models are prone to asthma induction or asthma aggravation, and a large number of neutrophils aggregate, resulting in airway inflammation, AHR and airway remodeling [33, 34]. At the same time, the use of an Nrf2 activator or an antioxidant stress treatment can improve symptoms, which confirms the importance of oxidative stress in neutrophilic asthma [35].

When a patient is infected with NTHi, innate immunity and adaptive immunity are activated in many ways, and inflammatory cells (including neutrophils, epithelial cells, macrophages, etc.) are activated [26]. NTHi, as an intracellular pathogen, can significantly increase the production of ROS in human fibroblasts, epithelial cells, macrophages and neutrophils after airway infection. ROS production was the highest in neutrophils and macrophages, and this production continued with prolonged NTHi infection. Moreover, a study also confirmed that ROS production was related to the formation of neutrophil extracellular traps (NETs), and the addition of DNase significantly inhibited the production of extracellular traps [36].

To kill NTHi, inflammatory cells develop respiratory bursts. During respiratory bursts, inflammatory cells release high concentrations of O_2_-, ·OH, HOCl/HOBr and H_2_O_2_, which can penetrate tissues and lead to increases in free radicals in surrounding tissues [32]. As a typical respiratory pathogen, NTHi has evolved a variety of defense mechanisms, such as catalase, peroxidase/glutamine enzyme and ferritin-like protein [37, 38]. This ability to resist oxidative stress is important for the colonization and pathogenicity of NTHi in the airway. The activation of excessive oxidative stress, such as through a signaling cascade, fails to kill NTHi but results in the imbalance of oxidants and antioxidants and may lead to cell damage through the oxidation of proteins, DNA and lipids, which has many adverse effects on the airway mucosa, including airway smooth muscle contraction, the induction of airway hyperresponsiveness, excessive mucus secretion, epithelial exfoliation, vascular exudation and airway remodeling [39, 40] (Fig. 2c). Furthermore, NTHi has a higher affinity for the damaged mucosa [41], leading to long-term colonization and chronic airway inflammation. There is a cycle of NTHi infection and oxidative stress: ROS induce the production of cytokines and chemokines by inducing oxidative stress-sensitive NF-κB transcription in bronchial epithelial cells, leading to further inflammation and more oxidative stress [42, 43]; this process further damages the airway mucosa and promotes the sustained colonization of NTHi. At the same time, sustained colonization promotes the progression of airway inflammation and the increase in oxidative stress. This process eventually leads to chronic airway inflammation and long-term, chronic colonization of NTHi.

Therefore, the level of oxidative stress in NTHi-infected asthmatic patients is high, which increases airway damage. Similarly, asthmatic patients with impaired airway barrier function are more vulnerable to oxidative stress caused by NTHi infection, which leads to the development of asthma.

#### Th1/Th2 imbalance

Numerous previous studies have confirmed that asthma is a chronic inflammatory airway disease mainly caused by the Th2 response. The excessive Th2 response leads to significantly increased levels of cytokines such as IL-14, IL-5, and IL-13, resulting in the aggregation of eosinophils in the airway, increased levels of IgE in the serum, and hyperresponsiveness of the airway [44] (Fig. 2d). It has been reported that the Th2 response was the main reaction in NTHi-colonized mice, and the Th1 response was inhibited, suggesting that chronic NTHi colonization may lead to an enhanced Th2 response. The Th1/Th2 imbalance hypothesis has been used to explain the pathogenesis of asthma in recent decades. In animal models of asthma, Th1/Th2 imbalances can be found, and some studies have tried to improve this imbalance to treat asthma [4, 45]. However, some clinical trials have yielded disappointing results, especially in the treatment of severe asthma, refractory asthma and glucocorticoid-resistant asthma [1, 46]. Over the past decade, the understanding of the pathogenesis of asthma has changed dramatically, from a single disease that is mediated by IgE and depends on Th2 cells to a more complex, heterogeneous disease. The Th1/Th2 imbalance hypothesis does not fully explain the pathogenesis of asthma, especially neutrophilic asthma [47, 48]. Th1/Th2 imbalances exist in neutrophilic asthma, but there must be other pathways beyond the Th2 pathway that play an important role in the pathogenesis of asthma, especially neutrophilic asthma. For example, strong Th17 responses can promote an increase in neutrophils in the airway and acute AHR, leading to acute asthma attacks [49]. In particular, the airway remodeling induced by Th17 cells is independent of the Th2 reaction and plays a crucial role in the pathogenesis of corticosteroid-resistant asthma and neutrophilic asthma [50]. It has been reported that NTHi-infected mice exhibit enhanced Th2 responses and suppressed Th1 responses, suggesting that NTHi infection may cause Th1/Th2 imbalances, but this is not the main mechanism for NTHi-related neutrophilic asthma [27].

#### Th17/Treg imbalance

Asthma is a chronic inflammatory disease of the airway. The T cell-mediated adaptive immune response plays a significant role in the occurrence and aggravation of neutrophilic asthma. Th17 and Treg cells are two important subsets of CD4+ T cells: Th17 cells can produce proinflammatory cytokines, of which IL-17 is a potent chemoattractant of neutrophils, which can mediate host defense against pathogens; Tregs play a negative regulatory role in immunity [51]. Research confirms that NTHi infection promotes the Th17 response and IL-17 secretion and inhibits the Treg response [4, 27]. As an inflammatory factor secreted by Th17 cells, IL-17A (one of the main members of the IL-17 family) plays a vital role in the occurrence of neutrophilic asthma, and the expression level of IL-17A is positively correlated with the severity of asthma [52, 53]. Th17/IL-17 can promote the activation of T cells, upregulate proinflammatory cytokines and chemokines, drive neutrophils to aggregate in the lungs, kill pathogenic bacteria, produce excessive airway neutrophilic inflammation, and damage the airway mucosa (Fig. 2e). This neutrophil inflammatory response can be reversed by enhancing Treg cell activity, suggesting that the imbalance of Th17/Treg cells may be a critical mechanism for the development of neutrophilic asthma.

In addition to causing airway neutrophilic inflammation, the Th17 response and elevated IL-17 can also promote airway remodeling, which is one of the most important features of asthma (Fig. 2e). Studies have shown that IL-17 can upregulate fibroblast growth factor and angiogenesis factor and can promote collagen synthesis [54]. At the same time, IL-17 is also believed to strongly promote the synthesis and secretion of the mucin MUC5AC (a major constituent of mucus) from goblet cells [55]. Moreover, IL-17 can also increase the proliferation and migration of human airway smooth muscle (ASM) cells through an ERK1/2-MAP kinase-dependent pathway [56], and Th17 cells can also inhibit ASM cell apoptosis [57]. Interestingly, bronchial fibroblasts obtained from asthmatic patients can specifically promote Th17 cell differentiation in vitro [58], which may indicate that the local airway environment of asthmatic patients is conducive to Th17 cell differentiation.

Additional studies have shown that Th17/Treg imbalances may also explain NTHi-related corticosteroid resistance in neutrophilic asthma. In vitro, dexamethasone can inhibit eosinophils, but not neutrophils, and can even promote the development of Th17 cells [50]. More detailed information on Th17/Treg imbalances and NTHi-related corticosteroid resistance will be discussed later in this article.

In conclusion, under physiological conditions, the proinflammatory response of Th17 cells and the anti-inflammatory response of Treg cells are in a state of balance, so that the immune response can eliminate pathogens without causing damage to the body. NTHi infection leads to the dominance of Th17 and its cytokines, can induce airway neutrophilic inflammation and can promote airway injury and airway remodeling, which is closely related to the development of neutrophilic asthma and may be a potential pathogenic mechanism of corticosteroid-resistant asthma.

#### Other potential mechanisms

The above mechanisms are possible mechanisms by which NTHi causes or promotes neutrophilic asthma. However, interestingly, in an ovalbumin (OVA)-sensitized model of asthma in mice, NTHi infection does not persist for more than 10 days but can promote asthma progression after 16 days [27]. Thus, the primary effect of this infection may be to induce persistent immune changes that persist even after the infection has been cleared, which behave synergistically with exposure to allergens to drive neutrophilic allergic airway diseases (AADs). Another mouse experiment also found that early exposure to NTHi significantly affected the incidence of anaphylactic airway diseases and the severity of AHR later in life [4]. A similar situation has been suggested in humans; children colonized with NTHi early in life were more likely to develop symptoms of asthma when they were older than children without detectable NTHi in the nasopharynx [59]. It has been speculated that NTHi colonization/infection may have a sustained impact on the immune system, contribute to the establishment of a proneutrophilic environment, and promote the occurrence and development of neutrophilic asthma in response to allergen challenges.

These results complement the hygiene hypothesis, which states that although high levels of microbial diversity in the environment are associated with a reduced risk of asthma, the colonization of specific dominant bacteria, such as NTHi, in the bronchi may be a risk factor for asthma.

### NTHi infection and corticosteroid-resistant asthma

At present, the symptoms of most asthmatic patients can be well controlled by treatment with inhaled corticosteroids combined with long-acting beta agonists, but some patients have a poor response to corticosteroids therapy; high-dose corticosteroids therapy is difficult to control, and the condition of repeated acute aggravation is called steroid resistance. Corticosteroid-resistant asthma is a serious global problem, leading to an enormous economic burden, and is one of the main causes of death in asthmatic patients. At the same time, corticosteroid resistance in asthma is a very complex problem. Current research shows that the mechanism of corticosteroid resistance may be related to genetic variation, epigenetics, asthma phenotypes, race and the airway microbiome [60].

NTHi is one of the most common bacteria isolated from asthmatic patients and is also associated with neutrophilic asthma and corticosteroid resistance in asthma. In vitro, *Haemophilus influenzae* has been shown to inhibit the corticosteroid response in asthmatic alveolar macrophages and peripheral blood mononuclear cells [61]. After NTHi infection, the TGF-β-activated kinase (TAK)-1/MAPK pathway was activated, and corticosteroid resistance was induced. Inhibition of the TAK-1 pathway can restore the sensitivity of cells to corticosteroids, suggesting that the development of corticosteroid resistance may be related to the TAK-1 pathway [62].

Another important mechanism of NTHi infection that promotes corticosteroid resistance is that NTHi infection leads to Th17/Treg homeostasis imbalances (as described in section 3.4), which leads to the occurrence of a neutrophilic asthma phenotype. IL-17 increased significantly after NTHi infection, which may be an important reason that NTHi infection promoted corticosteroid resistance. The transplantation of ova-treated Th17 cells into severely immunodeficient mice caused severe corticosteroid-resistant asthma [63], which confirmed the role of Th17 cells in corticosteroid resistance. IL-17 can counteract the effects of corticosteroids in human airway epithelial cells by inducing epigenetic changes and maintaining the secretion of inflammatory factors. It has been proven that IL-17 can affect the binding of glucocorticoid receptor (GR) to glucocorticoids (GS) by activating the phosphoinositide-3-kinase (PI3K) pathway and reducing histone deacetylase 2 (HDAC2) activity [64]. In addition, it was observed that IL-17 could increase the expression of GRb [65] in the peripheral blood cells of healthy donors. In patients with corticosteroid resistance, the imbalance of GRa/GRb may affect the binding of GR to CS, which may also lead to the occurrence of corticosteroid resistance.

In addition, another mechanism of NTHi colonization that promotes corticosteroid resistance may be related to oxidative stress. NTHi activates oxidative stress strongly after airway infection, as described in section 3.2, which causes airway smooth muscle contraction, the induction of airway hyperresponsiveness, excessive mucus secretion, epithelial exfoliation, vascular exudation and airway remodeling [25, 38]. Oxidative stress can also lead to further inflammation and damage the airway mucosa, promoting continuous colonization of NTHi and the invasion of other pathogens, which aggravate airway microbial disorders. Furthermore, oxidative stress can impair GR activity by lowering the activity of HDAC2, which has been shown to promote corticosteroid resistance in the lungs of patients with corticosteroid-resistant asthma [66, 67].

### Treatment of NTHi-related neutrophilic asthma

Presently, a large number of new studies on asthma treatment have emerged, providing many new ideas for clinical treatments. In particular, NTHi may be associated with the aggravation of neutrophilic asthma, so the targeted treatment of NTHi may effectively improve the symptoms of neutrophilic asthma.

#### Use of antibiotics

Using antibiotics to treat NTHi colonization is a direct and effective method, and macrolides are more suitable than other antibiotics. Studies have shown that long-term, low-dose azithromycin can regulate airway inflammation and inhibit NTHi-induced MUC5AC synthesis at the mRNA and protein levels by selectively inhibiting the activation of transcription factor activator protein-1 [68]. However, the long-term use of antibiotics results in the emergence of drug-resistant strains [12]. At the same time, an increasing number of studies have shown that children and pregnant women exposed to antibiotics are at increased risk of asthma later in life or in their newborn babies, and this risk is positively correlated with an increased antimicrobial spectrum and an increased antibiotic dosage [69–72]. The GINA report does not support the use of antibiotics for asthma, and it is recommended that antibiotics should not be routinely used to treat exacerbations of asthma unless there is definitive evidence of pulmonary infection (e.g., radiological evidence of pneumonia, fever or suppurative sputum) [2].

Therefore, when asthma is accompanied by NTHi infection, it is recommended to weigh the advantages and disadvantages and to decide whether to use antibiotics. The newly synthesized nonantibiotic macrolides have shown promise as prospective agents. These compounds can not only significantly improve macrophage phagocytosis, which is disturbed by NTHi, but can also regulate airway inflammation. At the same time, the antimicrobial activity of the newly synthesized nonantibiotic macrolides is significantly lower than that of antibiotics, thus significantly reducing the emergence of drug-resistant bacteria [73].

#### Other anti-inflammatory drugs

In addition to antibiotics, recent studies have found that some drugs targeting NTHi also have anti-inflammatory effects. Curcumin (from *Curcuma* plants) has a strong anti-inflammatory ability in otitis media caused by NTHi infection, and it can inhibit the NTHi-mediated upregulation of C-X-C motif chemokine 5 (CXCL5) expression by activating the inhibitor of nuclear factor kappa-B kinase kinase-β (IKKβ)-inhibitor kappa B (IκBα) and p38 MAPK pathways [74]. Studies have also shown that curcumin can inhibit airway inflammation and MUC5AC secretion in asthmatic mice [75]. Whether curcumin is effective in NTHi-induced neutrophilic asthma needs further study.

Resveratrol has long been considered an interesting drug for the treatment of various inflammatory diseases. Studies on its anti-inflammatory mechanism suggest that resveratrol increases the expression of MAPK phosphatase-1 through the camp/pka-dependent signaling pathway and inhibits NTHi-induced ERK1/2 phosphorylation. These results suggest that resveratrol has an anti-inflammatory effect after NTHi infection, so it has therapeutic potential in NTHi-related neutrophilic asthma.

Vitamin D is a unique nutrient that is multipotent, and it is believed to play a very important role in immune regulation. In addition, there is growing evidence that vitamin D also plays a protective role in lung diseases, such as allergic airway diseases, lung cancer and pulmonary fibrosis [76–78]. Vitamin D deficiency can increase the incidence of asthma and allergic airway diseases, and lung function can be improved by vitamin D supplementation, especially in patients with vitamin D deficiency [79]. Recent animal experiments have shown that 1,25-(OH)2D3 can regulate IL-17A at the transcriptional level through Runx1, thus alleviating airway inflammation in mice with neutrophilic asthma [80]. At the same time, it was observed in clinical trials that vitamin D can significantly improve steroid resistance in patients with severe asthma [81]. Therefore, it is speculated that vitamin D has potential value in the treatment of NTHi-related neutrophilic asthma.

#### Vaccines against NTHi

Epidemiological studies have shown that an increase in NTHi infection occurs after widespread vaccination with the *Haemophilus influenzae* type b conjugate vaccine [82]. Because NTHi can survive in cells and has many mechanisms to escape host immunity, routine therapy cannot eradicate NTHi colonization, and recurrent infection and chronic airway inflammation are common. Therefore, developing an NTHi vaccine may be key to preventing and treating NTHi-related diseases in the future [83].

#### Targeted therapy for IL-17

The importance of Th17/Treg homeostasis in the pathogenesis of neutrophilic asthma has been recognized by researchers. The NTHi-induced aggravation of neutrophilic asthma is closely related to an increase in IL-17. Interventions targeting Th17/Treg cells may be a new method for treating NTHi-related neutrophilic asthma, especially corticosteroid-resistant neutrophilic asthma [48, 49]. At present, many drugs targeting IL-17 are in clinical studies [84–86]. An antibody that neutralizes the human receptor for IL-17 (brodalumab, which can neutralize the activities of IL-17A, IL-17F and IL-25) has little effect on patients with mild to moderate asthma in clinical trials and has not achieved satisfactory results [87]. This may be related to the complex pathogenesis of neutrophilic asthma. In addition to Th17 cells and Treg cells, many immunoregulatory cells, such as Th2 cells, Th1 cells, innate lymphoid cells, dendritic cells, natural killer T cells, and TH9 cells, and many endogenous cytokines related to asthma, such as IL4, IL-5, IL13, IL22, and IL25, are also involved [1].

Neutrophilic asthma is regarded as a disease that has an exclusive Th17/Treg imbalance, which may be too simplified and seen only in extreme cases. In the clinic, various types of endogenous cytokines may overlap considerably and may be related to the severity of asthma. Therefore, more research and clinical work are needed on IL-17 targeted therapy.

#### Immunotherapy with oral NTHi

Earlier studies have found that sputum culture positivity and acute attack frequency were significantly rescued in moderate to severe COPD patients after the oral inactivation of NTHi [88]. Further studies have found that one of the mechanisms underlying the exacerbation of COPD is bacterial colonization, especially NTHi colonization, which results in neutrophilic inflammation in damaged airways, and this inflammation promotes the acute exacerbation of COPD [89]. In light of this hypothesis, oral immunotherapy with NTHi may enhance the delivery of intestinal antigens to Peyer’s patches, increase the number of T cells transported to the airway mucosa, and improve the efficiency of bacterial phagocytosis [89]. The protective effect of this treatment involves reducing the amount of NTHi in the damaged bronchi and protecting the airway. Based on this hypothesis, oral NTHi immunotherapy may also play a role in the aggravation of NTHi-induced neutrophilic asthma. To date, there have been no clinical studies on oral NTHi immunotherapy for neutrophilic asthma, which could be a good starting point for the treatment of neutrophilic asthma.

#### Nrf2 activators and antioxidants

Oxidative stress plays an essential role in asthma-related neutrophilic inflammation, AHR, increased mucus secretion and airway remodeling. It has been reported that asthmatic patients may have more difficulty coping with oxidative stress than healthy people, which may be closely related to the impairment of Nrf2 activity. Recent studies have also shown that Nrf2-driven glutathione-S-transferase may be a marker of susceptibility to asthma in humans [90]. Nrf2 agonists such as sulforaphane can inhibit neutrophilic airway inflammation and improve asthma symptoms in a mouse asthma model. Some classical antioxidants, such as vitamin E, can improve asthma symptoms by activating the Nrf2/heme oxygenase (HO)-1 pathway [91]. Combined with the current achievements in animal experiments, Nrf2 agonists and antioxidants may have great potential for application in NTHi-mediated neutrophilic asthma, but more evidence from different populations is needed for the clinical application of these treatments.

### Development potential and clinical applications of neutrophilic asthma drugs

The research and development of drugs for asthma and neutrophilic asthma are ongoing. The most recent clinical trial of asthma treatment is a phase IB clinical trial (EQUIP) of itolizumab. Itolizumab is a CD6-targeted antibody that has been suggested for the treatment of autoimmune diseases (such as psoriasis and arthritis). To date, the EQUIP clinical trial has not been completed, but itolizumab has been found to inhibit different effector T cell subtypes (including Th2 and Th17 cells), which play important roles in the pathogenesis of asthma; therefore, itolizumab may play a role in neutrophilic asthma [92].

The proinflammatory cytokine thymic stromal lymphopoietin (TSLP) is an epithelial cytokine produced by proinflammatory stimulation (such as that caused by pulmonary allergens, viruses and other pathogens) that plays a key role in the occurrence and persistence of airway inflammation. TSLP drives the release of downstream Th2 cytokines (including IL-4, IL-5 and IL-13) and activates various types of cells involved in non-Th2-driven inflammation. Tezepelumab is an anti-TSLP monoclonal antibody that specifically binds to human TSLP to block its interaction with its receptor complex. Tezepelumab is currently not listed in any country. In September 2017, AstraZeneca, a British pharmaceutical company, and Amgen, an AstraZeneca partner, published positive results of a phase 2B clinical trial conducted on tezepelumab use in patients with uncontrolled asthma, which showed that compared with that of patients taking a placebo, the annual asthma attack rate of patients receiving tezepelumab decreased by 71%. This result does not reflect the baseline eosinophil count and indicates the great potential of tezepelumab in the treatment of neutrophilic asthma [93, 94]. In October 2018, AstraZeneca and Amgen jointly announced that the US Food and Drug Administration (FDA) granted tezepelumab a breakthrough drug designation (BTD) for the treatment of patients with severe asthma with a noneosinophilic phenotype. Currently, tezepelumab is in phase III clinical development.

Neutrophilic asthma is usually characterized by corticosteroid resistance and severity; that is, the effect of corticosteroid treatment is unsatisfactory. The latest research shows that the new high-efficiency glucocorticoid ligands (such as vsg158) had a very high anti-inflammatory effect in animal asthma models, showing an effect 10-fold stronger than that of the widely used and effective asthma drug (fluticasone furoate, FF) and reversed corticosteroid resistance in the animal model of a corticosteroid-resistant type of malignant asthma. Although no clinical experiments were carried out, the research provided a new idea for the treatment of malignant asthma characterized by high mortality [95].

Neutrophils express chemokine receptors 1 and 2 (CXCR1 and 2) on the cell surface, interact with chemokines (such as interleukin 8), and recruit neutrophils to the site of inflammation or injury. Therefore, it has been suggested that the neutrophil chemokine receptor may be a potential therapeutic target for neutrophilic asthma. Azd5069 is a CXCR2 chemokine receptor antagonist that blocks the effect of interleukin 8. In a 6-month phase 2B clinical study on azd5069, although compared to the placebo, azd5069 reduced the mean blood neutrophil count, neither significant differences in the frequency of asthma attacks or symptoms nor changes in lung function were found. The definition of the neutrophilic asthma phenotype in this study was based on the fact that the blood neutrophil count was increased and the blood eosinophil count was reduced 0.5-fold. At 10^9^/L, the threshold eosinophil count may have been too high, resulting in some patients with an eosinophilic asthma subtype (combined with neutrophil elevation) being included in the trial, resulting in unsatisfactory treatment results. This outcome suggests that the blood neutrophil count may not be a good marker of neutrophilic asthma [96].

Recent research and development of drugs for neutrophilic asthma and related clinical experiments have provided some potential targets for the treatment of neutrophilic asthma, with some gratifying results. With continued research and an increased understanding of the potential mechanism of neutrophilic inflammation, it is imperative to use different drugs for different asthma subtypes, and more and larger clinical experiments are needed to promote progress in drug research.

## Conclusion

Neutrophilic asthma is a subtype of asthma that is closely related to the corticosteroid resistance of asthma and causes an enormous medical burden. The mechanism of airway inflammation in neutrophilic asthma is very complex; currently, its pathogenesis is not completely clear. Biological disorders of the lower airway have been shown to contribute to the worsening of neutrophilic asthma. NTHi is one of the most common bacteria in lower respiratory tract flora disorders; it is becoming increasingly clear that NTHi plays an important role in the progression of neutrophilic asthma and promotes the occurrence of corticosteroid resistance. In this study, the mechanisms of NTHi that promote the worsening of neutrophilic asthma were analyzed to explore the possible regulatory mechanisms in neutrophilic asthma, including hypersecretion of mucus, oxidative stress, Th1/Th2 imbalance and Th17/Treg imbalance. However, we should also note that these studies are incomplete, and many issues remain poorly understood, such as the study of how Th17 cells and Th2 cells cooperate in neutrophilic asthma and how NTHi infection cooperates with or antagonizes other bacterial and viral infections in the lower airway disorders of neutrophilic asthma; these topics deserve further study.

In view of the role of NTHi in neutrophilic asthma, many therapeutic measures against NTHi in asthmatic patients are being explored at present, such as the use of antibiotics, the use of other anti-inflammatory drugs, the development of an NTHi vaccine, IL-17 targeted therapy, oral NTHi immunotherapy, and antioxidants for the increased airway oxidative stress caused by NTHi. Clinical trials for drug research and development for neutrophilic asthma are also in progress, such as trials of itolizumab and tezepelumab, and some satisfactory results have been obtained, showing the potential of targeted treatment for neutrophilic asthma.

In conclusion, NTHi infection provides a new target for studying the pathogenesis and treatment of neutrophilic asthma. It is believed that with additional research, the pathogenesis of neutrophilic asthma will be further elucidated, and more targeted drugs will be designed for the specific phenotype and endotype of neutrophilic asthma.

## Data Availability

All data generated or analyzed during this study are included in this published article.

## Abbreviations

ICS: inhaled corticosteroids
GINA: Global Initiative for Asthma
IL: interleukin
AHR: airway hyperreactivity
NTHi: Nontypeable *Haemophilus influenzae*
LOSs: lipooligosaccharides
PAFR: platelet activating factor receptor
COPD: chronic obstructive pulmonary disease
MAPK: mitogen-activated protein kinase
NF-κB: nuclear factor-kappa B
ROS: oxygen species
Nrf2: Nuclear factor erythroid 2-related factor 2
NETs: neutrophil extracellular traps
ASM: airway smooth muscle
OVA: ovalbumin
AADs: allergic airway diseases
TAK: TGF-β-activated kinase
GR: Glucocorticoid receptor
GS: Glucocorticoids
PI3K: Phosphoinositide-3-kinase
HDAC2: histone deacetylase 2
CXCL5: C-X-C motif chemokine 5
IKKβ: inhibitor of nuclear factor kappa-B kinase-β
IκBα: inhibitor kappa B
HO: heme oxygenase
TSLP: thymic stromal lymphopoietin;
